# Complete Remission of Advanced Adrenocortical Cancer Following Mitotane Monotherapy: A Case Report and Literature Review of Predictive Markers

**DOI:** 10.3389/fonc.2021.680853

**Published:** 2021-05-11

**Authors:** Judit Tőke, Zsuzsanna Jakab, Júlia Stark, Gergely Huszty, Péter Reismann, Miklós Tóth

**Affiliations:** ^1^ Department of Internal Medicine and Oncology, ENDO-ERN HCP, Semmelweis University, Budapest, Hungary; ^2^ Department for Transplantation and Surgery, Semmelweis University, Budapest, Hungary

**Keywords:** adrenocortical cancer, complete remission, predictive markers, mitotane, monotherapy

## Abstract

Mitotane has been used for the treatment of adrenocortical cancer (ACC) for over 50 years. Despite its widespread use both in monotherapy and in combination with chemotherapeutics, our knowledge of its mechanism of action and therapeutic efficacy is scarce. The number of patients with advanced ACC who have achieved complete remission documented by detailed clinical data is below ten. We report a case of a 64-year-old woman with a non-functional ACC. Histological examination showed vascular invasion, Ki67 of 10% and a mitotic count of 3/10 high-power field. Immunohistochemistry revealed p53 positivity. Pathological TNM grade was reported as T2N0M0, ENSAT stage 2. Nine months after the initial diagnosis, re-staging CT revealed multiple peritoneal nodules, lymph node and kidney metastases confirmed by histologic examination. Mitotane monotherapy was started with a maintenance dose between 2.0 and 2.5 grams/day. Partial remission was established at six months. Subsequently, for another 12 months, each of the three-monthly CT scans confirmed complete remission. Nineteen months after the initiation of mitotane, an unexpected sudden death occurred. A detailed autopsy work-up, performed in the full awareness of oncological history, confirmed complete remission. The authors review the molecular biomarkers and clinical features reported as predictors of response to mitotane monotherapy.

## Introduction

Nowadays, mitotane is the only compound registered to treat adrenocortical cancer (1). Despite its use of over 50 years, the exact mechanism of action is mostly unknown (2). Mitotane is most frequently used in combination with chemotherapeutics. For recurrent or advanced adrenocortical cancer, etoposide-doxorubicin-cisplatin therapy combined with mitotane (EDP-M) is recommended as the first-line treatment of choice. In second-line settings, gemcitabine plus capecitabine or streptozocin could be possible options with continued mitotane treatment (3). In patients with low tumour load or poor performance status, mitotane can be initiated as monotherapy (4). Although mitotane is not registered for adjuvant purposes, it is used with increasing frequency in the adjuvant setting (5–7).

The efficacy of EDP-M is limited in general; however, favourable response is obtained in a few cases. Terzolo et al. reported their single-centre experiences with 180 metastatic ACC patients treated with EDP-M therapy over 20 years. Four patients (2.2% of all) exhibited progression-free survival for five years. Two patients showed complete remission after EDP-M chemotherapy (8). In another single-institution series, surgery identified complete pathological response in 4 (7%) out of 58 consecutive metastatic ACC patients following EDP-M. None of them had recurred at the last follow-up (9).

Despite its widespread use in the therapy of ACC, we have limited knowledge about its efficacy as monotherapy. The most extensive study to date reported experiences with 127 patients from a German multicentre study (10). In this retrospective analysis, 26 patients (20,5%) exhibited objective response; three of them had a complete response (10). Another study from the Memorial Sloan-Kettering Cancer Center from the period between 1989 and 2015 showed that only 4 out of the 36 patients (11%) had an objective response to mitotane monotherapy; however, 3 of them showed complete response (11).

Concerning complete remission achieved by mitotane monotherapy in patients with advanced-stage ACC, there is a remarkable paucity of reported patients. El Ghorayeb et al. summarized all the nine adult patients with advanced ACC having achieved complete remission with mitotane monotherapy, published between 1974 and 2014 (12). Since this publication, we are aware of six other cases reported without further details in the two aforementioned retrospective clinical studies (10, 11). Besides reporting a new patient, we review the molecular and clinical predictors of response to mitotane monotherapy in advanced ACC patients.

## Case Description

**Figure 1:**
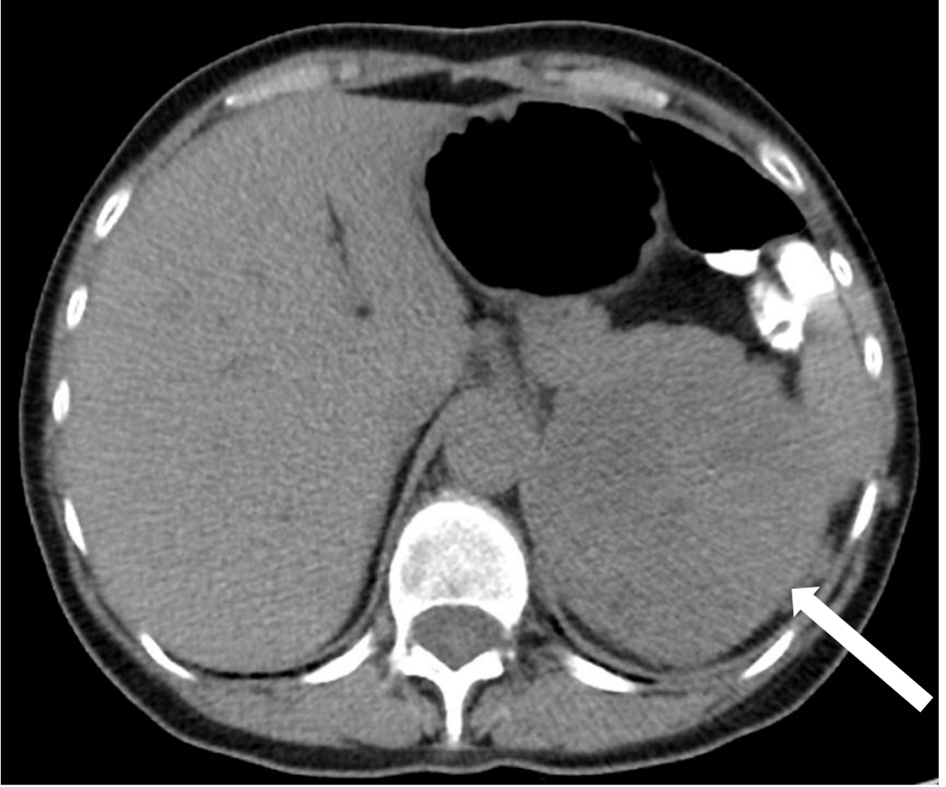
Left adrenal tumour, 9.5 cm in its largest diameter. Unenhanced computed tomographic scan. Native density 40 Hounsfield unit.

A 64-year-old woman was referred to our adrenocortical cancer referral centre following left adrenalectomy. The adrenal tumour, 9.5 cm in its largest diameter, was detected incidentally during investigations for an unexplained rash (**Figure 1**). Preoperative hormonal measurements including plasma cortisol, aldosterone, ACTH, and plasma renin activity resulted in the diagnosis of a non-functional adrenal tumour. Chest and abdominal computed tomography revealed discrete peritumoral adipose tissue infiltration without pathological lymph nodes or distant metastases. The native density of the tumour on CT was +40 Hounsfield unit. Histological examination showed vascular invasion, Ki67 of 10% and a mitotic count of 3/10 high-power field. Immunohistochemistry revealed p53 positivity. Pathological TNM grade was reported as T2N0M0, ENSAT stage 2.

**Figure 2:**
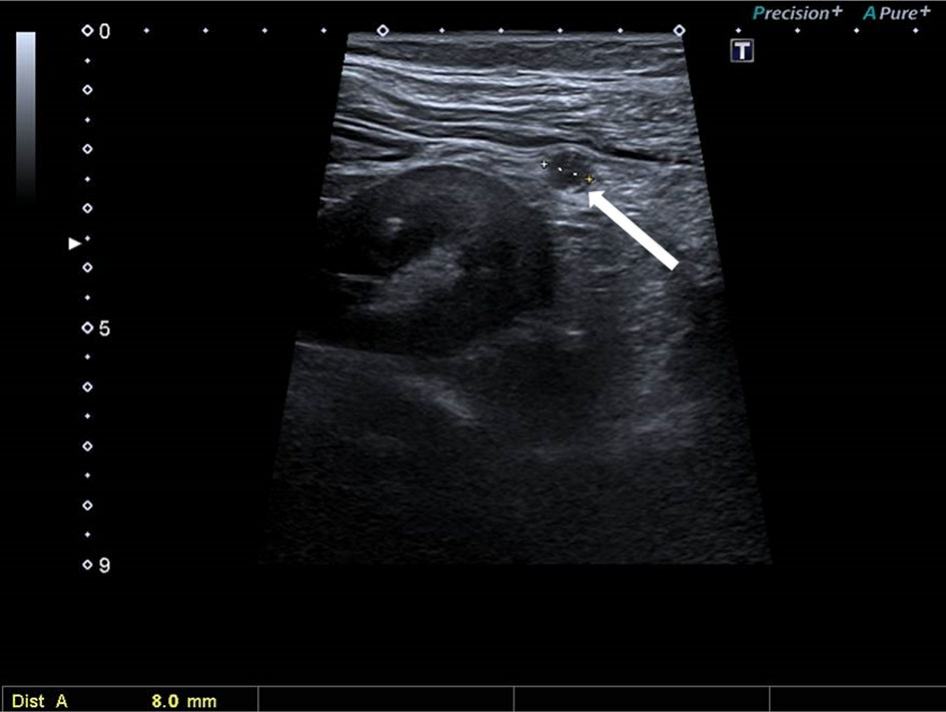
8 mm metastasis within the left perirenal fat. Ultrasonographic image.

**Figure 3:**
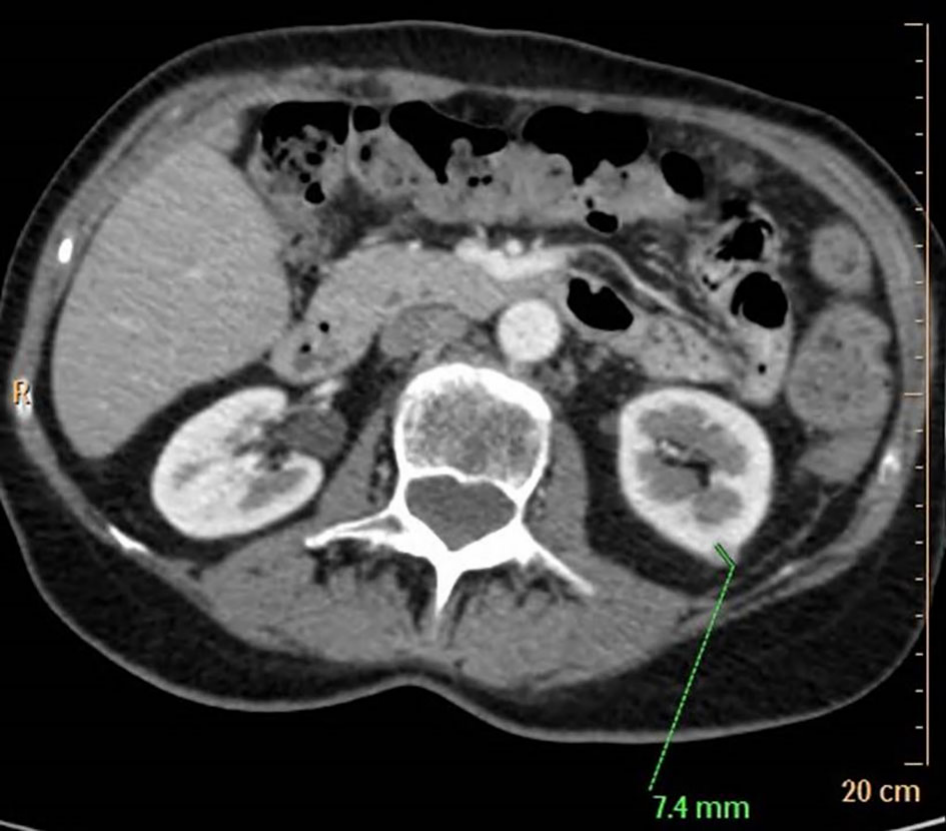
7.4 mm metastasis in the left renal cortex. Contrast-enhanced computed tomographic scan.

2,5 months following adrenalectomy, the tumour bed was irradiated using a Varian Clinac iX 6 MV photon beam instrument with a total dose of 50.5 Gy. The postoperative 3-and 6-month re-staging CT did not show any tumour lesion. Nine months after the initial diagnosis, abdominal ultrasonography and re-staging CT revealed multiple peritoneal nodules raising the suspicion of peritoneal carcinomatosis, a periventricular lymph node, and a left kidney metastasis (**Figures 2** and **3**). The maximal tumoral diameter was 12 mm. Laparoscopic tissue sampling from the ascending colon and sigmoid intestine resulted in the histological diagnosis of peritoneal carcinomatosis from ACC. Therefore, twelve months following adrenalectomy, mitotane monotherapy was started according to a high-dose regimen (13). Mitotane plasma levels were monitored by the Lysosafe service (www.lysosafe.com). Therapeutic mitotane concentration was achieved within two months. Mitotane, with a maintenance dose between 2.0 and 2.5 grams/day, was well tolerated during the whole course of its administration. The patient was substituted with hydrocortisone, 30 mg/day. She was educated for signs and symptoms of adrenal failure and supplied with an emergency card and parenteral hydrocortisone KIT.

Re-staging CT performed three months following the initiation of mitotane therapy revealed stable disease, while partial remission was reported at six months. After that, each three-monthly CT confirmed complete remission.

Two and a half years following the adrenalectomy and nineteen months after the initiation of mitotane, the lone-living patient was found dead in her flat. A detailed pathological work-up, performed in the full awareness of oncological history, confirmed complete remission. Special attention was paid to the peritoneal surfaces and the liver, which was sliced into 5-mm slices. Lesions suspected of metastatic disease were not found anywhere. The autopsy could not give a definite answer regarding the cause of death. Because of the influenza epidemic at that time, a viral infection could be suspected as a cause of death. Other alternatives as possible explanations for the unexpected sudden death include adrenal insufficiency and long Q-T syndrome caused by mitotane therapy.

According to our in-house ACC registry, out of 48 patients with advanced ACC, 15 patients were initially treated with mitotane monotherapy. The patient reported here is the only one who achieved complete remission on mitotane monotherapy.

## Discussion

Mitotane causes selective damage to adrenocortical cells, acting primarily by the disruption of mitochondria and activating apoptosis (14). Regarding the potential molecular biomarkers of the efficacy of mitotane therapy, data are scarce. Maintaining serum mitotane levels in the target range of 14 to 20 mg/L is a strong predictor of effectiveness (15). However, we have several hints that objective response to mitotane can be achieved with lower plasma mitotane concentrations (10, 11). The human cytochrome P450 2W1 enzyme (CYP2W1) was suggested to be involved in the metabolism of mitotane. CYP2W1 immunoreactivity was associated with longer overall survival and time to progression in ACC patients treated with mitotane monotherapy (16). A recent multicentre study suggested that a specific combination of single nucleotide polymorphisms of two mitochondrial enzymes (CYP2W1 and CYP2B6) may predict therapeutic response to mitotane monotherapy (17). Next-generation sequencing proved unsuccessful in predicting response to mitotane monotherapy in two out of the three complete responders to mitotane (11). Mitotane is an inhibitor of sterol-O-acyl transferase 1 (SOAT1), which was postulated to be a key molecular target of mitotane. However, SOAT1 expression has not been correlated with clinical endpoints in a study with ACC patients on mitotane monotherapy (18). Despite numerous efforts, none of the studies established clinically useful biomarkers having the potential for predicting response to mitotane therapy. The results of these studies are summarized in **Table 1**.

**Table 1:** Molecular markers tested so far for a response to mitotane monotherapy in patients with advanced ACC.

Presumed biomarker	No of the tested patients	Result	Reference
DNA repair enzymes RRM1 and ERCC1	92	RRM1 expression was associated with DFS and OS	Volante et al. (19)
Germline DNA	2	NGS was negative in complete responders	Reidy-Lagunes et al. (11)
CYP2W1 expression	25	CYP2W1 predicts response to mitotane	Ronchi et al. (16)
SNPs (CYP2W1*2, CYP2W1*6, CYP2B6*6 and CYP2B6 rs4803419)	182	CYP2W1*6 and CYP2B6*6 may predict individual response	Altieri et al. (17)
Sterol-O-acyl transferase 1 (SOAT1)	231	no correlation with clinical endpoints	Weigand et al. (18)

DFS, disease-free survival, ERCC1: excision repair cross-complementation group 1; NGS, next-generation sequencing; OS, overall survival; RRM1, Ribonucleotide reductase large subunit 1; SNP, single nucleotide polymorphism.

Similarly to potential biochemical markers, to date, we do not have firm clinical markers predicting response to mitotane (12). Two extensive retrospective studies were published with mitotane monotherapy in their focus (10, 11), and only one paper summarized individual case reports to date (12). **Table 2** updates the main clinicopathological features of each patient reported with complete response to mitotane monotherapy. In these publications, a common clinical parameter predicting response to mitotane was the tumour burden itself, expressed either as the number of sites involved or tumoral diameter or number of tumoral lesions. Late versus early recurrence of advanced disease (cut-off at 360 days) proved to be another highly significant clinical parameter (10). According to these clinical reports, hormonal activity of the primary tumour is not an important feature influencing response to mitotane as complete remission could be achieved both in functioning and in non-functioning adrenocortical cancers. The metabolic response to mitotane on FDG-PET scan was suggested to be a potential radiologic predictor of response to mitotane monotherapy  (12).

**Table 2:** Clinicopathological features of adult patients with metastatic adrenocortical cancer reported as complete responders to mitotane monotherapy.

Reference	No of patients reported with CR to mitotane	Timing of mitotane initiation	Tumour burden at mitotane initiation	Hormonal excess
Megerle et al. (10)	3	≥360 days since initial diagnosis (3/3)	< 10 tumor lesion (2/3)	Cortisol (1/3)
			≥ 10 tumor lesion (1/3)	
Reidy-Lagunes et al. (11)	3	ND	Tumors in one site with low (< 3 cm)	Nonfunctional (3/3)
			tumor volume (3/3)	
El Ghorayeb et al. (12)	9	At initial diagnosis (6/9)	ND	Nonfunctional (3/9)
		2 years since initial diagnosis (1/9)		Cortisol (1/9)
		ND (2/9)		Androgens (3/9)
				Cortisol and androgens (2/9)
Our patient, 2021	1	1 year since initial diagnosis (1/1)	< 10 tumor lesion, low tumor burden (maximum tumor diameter 12 mm)	Non-functional (1/1)

ND, no data.

Concerning the suggested clinical predictors of a favourable response to mitotane, our patient fulfilled three out of the four parameters listed in **Table 3**. She was in good condition at the tumour’s recurrence; she had a non-functional tumour and had a low tumour burden with less than ten lesions at recurrence. Only one criterion was not fulfilled; namely, her tumour relapsed after 270 days.

**Table 3:** Clinical parameters suggested predicting favourable response to mitotane monotherapy in patients with advanced ACC.

	Reidy-Lagunes et al., 2017 (11)	Megerle et al., 2018 (10)
Endocrine activity	non-functional tumours probably respond better	not a predictive factor
Performance status	Patients with ECOG 0-1 probably respond better	not investigated
Tumour burden	disease limited to one site	< 10 tumoral lesions
	low volume disease (< 3 cm)	
Timing of recurrence	not investigated	delayed (>360 days) advanced recurrence

Our case presentation has limitations. First, the cure of peritoneal carcinomatosis was initially diagnosed and followed with computed tomography scans which could not imply a complete pathological response in vivo. Nevertheless, the autopsy findings, including the inspection of the peritoneal surfaces, should be considered as strong indicators of complete remission. In addition, the follow up of this patient was short (19 months) due to the occurrence of the sudden death.

The optimal length of mitotane monotherapy following achievement of complete response is unknown. Within El Ghorayeb’s compilation of patients with complete remission, disease-free survivals were between 4 and 25 years. Regarding the length of mitotane therapy following CR, we do not have any guidance. In some patients, mitotane was administered lifelong; however, long-lasting disease-free intervals were reported in patients with discontinued mitotane therapy.

## Concluding Remarks

The few reported cases of patients with advanced ACC achieving complete remissions on mitotane monotherapy are strong pieces of evidence for the therapeutic efficacy of mitotane. Molecular biomarkers predicting the success of mitotane are desperately needed.

## Data Availability Statement

The original contributions presented in the study are included in the article/supplementary material. Further inquiries can be directed to the corresponding author.

## Author Contributions

JT and MT wrote the first draft of the manuscript. JT, ZJ, PR, and MT participated in the diagnosis and treatment of the patient, and providing follow-up. GH, PR, and JS acquired clinical data. All authors contributed to the article and approved the submitted version.

## Conflict of Interest

The authors declare that the research was conducted in the absence of any commercial or financial relationships that could be construed as a potential conflict of interest.

## Abbreviations

ACC, adrenocortical carcinoma; ACTH, adrenocorticotropic hormone; CT, computed tomography; FDG-PET-CT, fluoro-deoxyglucose positron emission tomography; ENDO-ERN HCP, European Reference Network on Rare Endocrine Conditions, Health Care Provider.

## References

[1] Paragliola RM Corsello A Locantore P Papi G Pontecorvi A Corsello SM. Medical Approaches in Adrenocortical Carcinoma. Biomedicines (2020) 8(12). 10.3390/biomedicines8120551 PMC7760807 33260476

[2] Fassnacht M Kroiss M Allolio B. Update in Adrenocortical Carcinoma. J Clin Endocrinol Metab (2013) 98(12):4551–64. 10.1210/jc.2013-3020 24081734

[3] Fassnacht M Assie G Baudin E Eisenhofer G de la Fouchardiere C Haak HR. Adrenocortical Carcinomas and Malignant Phaeochromocytomas: ESMO-EURACAN Clinical Practice Guidelines for Diagnosis, Treatment and Follow-Up. Ann Oncol (2020) 31(11):1476–90. 10.1016/j.annonc.2020.08.2099 32861807

[4] Else T Kim AC Sabolch A Raymond VM Kandathil A Caoili EM. Adrenocortical Carcinoma. Endocr Rev (2014) 35(2):282–326. 10.1210/er.2013-1029 24423978 PMC3963263

[5] Puglisi S Calabrese A Basile V Pia A Reimondo G Perotti P. New Perspectives for Mitotane Treatment of Adrenocortical Carcinoma. Best Pract Res Clin Endocrinol Metab (2020) 34(3):101415. 10.1016/j.beem.2020.101415 32179008

[6] Terzolo M Angeli A Fassnacht M Daffara F Tauchmanova L Conton PA. Adjuvant Mitotane Treatment for Adrenocortical Carcinoma. N Engl J Med (2007) 356(23):2372–80. 10.1056/NEJMoa063360 17554118

[7] Terzolo M Baudin AE Ardito A Kroiss M Leboulleux S Daffara F. Mitotane Levels Predict the Outcome of Patients With Adrenocortical Carcinoma Treated Adjuvantly Following Radical Resection. Eur J Endocrinol (2013) 169(3):263–70. 10.1530/eje-13-0242 23704714

[8] Terzolo M Daffara F Ardito A Zaggia B Basile V Ferrari L. Management of Adrenal Cancer: A 2013 Update. J Endocrinol Invest (2014) 37(3):207–17. 10.1007/s40618-013-0049-2 24458831

[9] Laganà M Grisanti S Cosentini D Ferrari VD Lazzari B Ambrosini R. Efficacy of the EDP-M Scheme Plus Adjunctive Surgery in the Management of Patients With Advanced Adrenocortical Carcinoma: The Brescia Experience. Cancers (Basel) (2020) 12(4). 10.3390/cancers12040941 PMC7226395 32290298

[10] Megerle F Herrmann W Schloetelburg W Ronchi CL Pulzer A Quinkler M. Mitotane Monotherapy in Patients With Advanced Adrenocortical Carcinoma. J Clin Endocrinol Metab (2018) 103(4):1686–95. 10.1210/jc.2017-02591 29452402

[11] Reidy-Lagunes DL Lung B Untch BR Raj N Hrabovsky A Kelly C. Complete Responses to Mitotane in Metastatic Adrenocortical Carcinoma-a New Look At an Old Drug. Oncologist (2017) 22(9):1102–6. 10.1634/theoncologist.2016-0459 PMC5599197 28559412

[12] El Ghorayeb N Rondeau G Latour M Cohade C Olney H Lacroix A. Rapid and Complete Remission of Metastatic Adrenocortical Carcinoma Persisting 10 Years After Treatment With Mitotane Monotherapy: Case Report and Review of the Literature. Med (Baltimore) (2016) 95(13):e3180. 10.1097/md.0000000000003180 PMC4998541 27043680

[13] Fassnacht M Dekkers OM Else T Baudin E Berruti A de Krijger R. European Society of Endocrinology Clinical Practice Guidelines on the Management of Adrenocortical Carcinoma in Adults, in Collaboration With the European Network for the Study of Adrenal Tumors. Eur J Endocrinol (2018) 179(4):G1–g46. 10.1530/eje-18-0608 30299884

[14] Poli G Guasti D Rapizzi E Fucci R Canu L Bandini A. Morphofunctional Effects of Mitotane on Mitochondria in Human Adrenocortical Cancer Cells. Endocr Relat Cancer (2013) 20(4):537–50. 10.1530/erc-13-0150 23722227

[15] Hermsen IG Fassnacht M Terzolo M Houterman S den Hartigh J Leboulleux S. Plasma Concentrations of O,P’ddd, O,P’dda, and O,P’dde as Predictors of Tumor Response to Mitotane in Adrenocortical Carcinoma: Results of a Retrospective ENS@T Multicenter Study. J Clin Endocrinol Metab (2011) 96(6):1844–51. 10.1210/jc.2010-2676 21470991

[16] Ronchi CL Sbiera S Volante M Steinhauer S Scott-Wild V Altieri B. CYP2W1 is Highly Expressed in Adrenal Glands and is Positively Associated With the Response to Mitotane in Adrenocortical Carcinoma. PloS One (2014) 9(8):e105855. 10.1371/journal.pone.0105855 25144458 PMC4140842

[17] Altieri B Sbiera S Herterich S De Francia S Della Casa S Calabrese A. Effects of Germline CYP2W1*6 and CYP2B6*6 Single Nucleotide Polymorphisms on Mitotane Treatment in Adrenocortical Carcinoma: A Multicenter ENSAT Study. Cancers (Basel) (2020) 12(2). 10.3390/cancers12020359 PMC7072643 32033200

[18] Weigand I Altieri B Lacombe AMF Basile V Kircher S Landwehr LS. Expression of SOAT1 in Adrenocortical Carcinoma and Response to Mitotane Monotherapy: An ENSAT Multicenter Study. J Clin Endocrinol Metab (2020) 105(8):2642–53. 10.1210/clinem/dgaa293 32449514

[19] Volante M Terzolo M Fassnacht M Rapa I Germano A Sbiera S. Ribonucleotide Reductase Large Subunit (RRM1) Gene Expression May Predict Efficacy of Adjuvant Mitotane in Adrenocortical Cancer. Clin Cancer Res (2012) 18(12):3452–61. 10.1158/1078-0432.ccr-11-2692 22547773

